# Genetic Risk Factors of Systemic Lupus Erythematosus in the Malaysian Population: A Minireview

**DOI:** 10.1155/2012/963730

**Published:** 2011-09-20

**Authors:** Hwa Chia Chai, Maude Elvira Phipps, Kek Heng Chua

**Affiliations:** ^1^Jeffrey Cheah School of Medicine and Health Sciences, Monash University, Sunway Campus, 46150 Selangor Darul Ehsan, Malaysia; ^2^Department of Molecular Medicine, Faculty of Medicine, University of Malaya, 50603 Kuala Lumpur, Malaysia

## Abstract

SLE is an autoimmune disease that is not uncommon in Malaysia. In contrast to Malays and Indians, the Chinese seem to be most affected. SLE is characterized by deficiency of body's immune response that leads to production of autoantibodies and failure of immune complex clearance. This minireview attempts to summarize the association of several candidate genes with risk for SLE in the Malaysian population and discuss the genetic heterogeneity that exists locally in Asians and in comparison with SLE in Caucasians. Several groups of researchers have been actively investigating genes that are associated with SLE susceptibility in the Malaysian population by screening possible reported candidate genes across the SLE patients and healthy controls. These candidate genes include *MHC* genes and genes encoding complement components, TNF, Fc*γ*R, T-cell receptors, and interleukins. However, most of the polymorphisms investigated in these genes did not show significant associations with susceptibility to SLE in the Malaysian scenario, except for those occurring in *MHC* genes and genes coding for TNF-*α*, IL-1*β*, IL-1RN, and IL-6.

## 1. Introduction

Systemic lupus erythematosus (SLE) is the prototypical autoimmune disease that is characterized by autoantibody production, complement activation, and immune complex deposition leading to diverse clinical manifestations and target tissue damage. The prevalence of SLE is estimated to be between 40 and 400 cases per 100,000 individuals [1]. While the precise etiology of SLE still remains vague, genetic predisposition and environmental and hormonal factors are deemed to play important roles in its pathogenesis. Severity, acquisition risk, and clinical manifestations of this disease can vary by ethnicity, geography, and sex, with a prevalence that is higher in women during their childbearing ages and some non-European populations such as African Americans, Hispanics, and Asians [2, 3]. 

In Asians, the prevalence of SLE generally falls within 30–50/100,000 individuals. SLE is more frequent among Chinese communities in Asia than it is in India and tropical Africa [4]. Malaysia is a multiracial country. In the peninsular, the Malays (55.1%), Chinese (24.3%), and Indians (7.4%) represent the largest ethnic groups. A prevalence of 43/100,000 individuals in Malaysia has been reported [5, 6]. Likewise, Chinese have the highest prevalence of SLE in Malaysia (57/100,000), followed by Malays (33/100,000) and Indians (14/100,000) [7, 8]. The overall 5-year and 10-year survival rates were reported as 82% and 70%, respectively [5], whereas the overall mortality rate was 20.2% [9]. Renal involvement is highest among the Malaysian patients [5]. However, the major cause of death in Malaysian SLE patients was reported to be from infection [9].

Pathogenesis of SLE is associated with functional deficiency of multiple immunologic components, including the innate immune system, altered immune tolerance mechanisms, hyperactivation of T and B cells, reduced ability of immune complexes and apoptotic cell clearance, and defects in multiple immune regulatory networks [10]. The failure of these mechanisms could be due to the influence of variants within SLE susceptibility genes. To date, many different genes have been found to contribute to disease susceptibility. In a small proportion of patients (<5%), a single gene could become the key player for this disease [11]; however, multiple genes have been implicated in most patients. It is estimated that at least four susceptibility genes or loci are needed for the development of the disease [12]. The susceptibility genes most extensively studied are within the major histocompatibility complex (MHC). It is believed that human leukocyte antigen (HLA) class II gene variants are very important. The introduction of genome-wide association studies (GWASs) has not only helped us to support the findings from previous candidate gene studies, but also unveiled many other novel genetic loci that may be important. Candidate genes that have been recently discovered can be clustered into three main groups: (i) *IRF5*, *STAT4*, *TNFAIP3,* and *TREX1 *which are involved in innate immune response including TLR/interferon signalling pathway; (ii) *HLA-DR*, *PTPN22*, *PDCD1*, *LYN*, *BLK,* and *BANK1* which are involved in immune signal transduction of B, T, and antigen-presenting cells; (iii) *C2*, *C4*, *FCGRs*, *CRP,* and *ITGAM* which are involved in immune complex clearance mechanism [13, 14]. 

The genetic information obtained from GWAS has allowed many researchers to investigate specific variants for particular genetic loci using a variety of approaches such as RFLP-PCR, tetra-primer ARMS-PCR, and real-time genotyping PCR. This in turn has enabled the replication of these experiments and confirmed those associated polymorphisms with SLE in different populations. Genetic heterogeneity is common among populations in SLE, especially between Caucasians and Asians. For instance, *PTPN22,* which demonstrated significant association with SLE in Caucasians, was not found to be associated with some ethnicities in Asia [15]. The identification of genetic heterogeneity may enhance our understanding of mechanisms that lead to SLE pathogenesis in certain populations and subsequently may permit more precise diagnosis, prognosis, and treatment for the patients. 

In Malaysia, researchers have been actively looking into the genetic risk factors of SLE in the multiracial population for the past 15 years. These efforts have generated a considerable amount of data that have been useful contributions to enriching global statistics and knowledge of SLE. PCR-based methods were mainly used in these studies. In this paper, the association of several candidate susceptibility genes with SLE in Malaysian population will be discussed and summarized (Table 1). In addition, genetic heterogeneity in SLE susceptibility observed in different ethnicities will be discussed.

## 2. Candidate Genes

### 2.1. Major Histocompatibility Complex Genes

The major histocompatibility complex (MHC), which contains human leukocyte antigen (*HLA*) genes, is a large genomic region located on chromosome 6. HLA antigens and genes have long been associated with SLE, and this can be dated back to 1971, when Grumet et al. [33] reported a possible relationship. Of the several classes of HLA, HLA class II genes seem particularly important in SLE. They encode cell-surface antigen-presenting proteins that present antigens to T cells and in turn stimulate the multiplication of T-helper cells and production of antibodies by B cells. HLA class II genes have also been associated with the presence of certain autoantibodies such as anti-Sm, anti-Ro, anti-La, anti-nRNP, and anti-DNA antibodies, which have been useful biomarkers in SLE diagnosis. HLA-DR2 has been reported to be consistently associated with SLE in both Caucasian and Asian populations [34, 35]. HLA class III genes, particularly those encoding complement components C2 and C4, may also confer increased risk for SLE in different ethnicities

In Malaysia, Azizah et al. [16] reported significant association of HLA-DR2, -DQB1∗0501, and -DQB1∗0601 with SLE in Malays. A significant positive association of DR2 and DQB1∗0501 with renal involvement and DR8 with alopecia in Malays was also described in their study. For the investigation of the role of *HLA *genes in autoantibody expression, they found significant association of DQB1∗0601 with anti-Sm/RNP, DR2 with anti-Ro/La, and DR2, DRB1∗0501 and ∗0601 with anti-dsDNA. The same group of researchers also carried out similar study on Chinese population and suggested that DQB1∗0102, DQB1∗0501, ∗0601, and DPB1∗0901 were significantly associated with SLE [17]. Clinically, a strong association of DR2 and DQA1∗0301 with renal involvement and DQA1∗0102 with alopecia was reported. In contrast to Malays, DQA1∗0102 and DQA1∗0301 were observed to be strongly associated with anti-Ro/La and anti-dsDNA, respectively, in Chinese. Earlier on, Doherty et al. [18] reported that HLA-DRw15 and DQw1 were observed to be significantly associated with SLE among Southern Chinese in Malaysia and most prevalent in patients with lupus nephritis and cutaneous manifestation. 

A recent comprehensive study conducted by Mohd-Yusuf and coworkers [19] in Malaysia revealed that HLA A∗1101, 1102, DRB5∗01-02, DQB1∗05, DRB3∗0101, 0201, 0202, 0203, 0301, and DQB1∗0301, 0304 were significantly associated with SLE in Malaysians. In addition, DRB1∗0701 and DRB4∗0101101, 0102, 0103 alleles were significantly increased in the Malay SLE patients, whilst DRB1∗1601-1606 (DR2 subtype) and DRB5∗0101, 0102, 0201, 0202, 0203 alleles were significantly higher in Chinese SLE patients. The investigation revealed that these two different sets of DR alleles may be specific and representative for the two ethnic groups in this SLE cohort and that DQB1∗05 could be the common HLA susceptibility allele in the Malaysian SLE population.

### 2.2. Complement Components

The complement system is mainly involved in innate immunity, whereby it helps to remove cellular debris from foreign and apoptotic cells. The links between complement system activity with SLE have been reported since the 1980s. Mutant C4 genes have been mostly reported in Caucasian families, but are still uncommon in other populations. Apart from Caucasians, the presence of C4A null allele (C4aQ0) was also observed in Chinese and Japanese with SLE by Dunckley et al. [36]. In the Malaysian scenario, none of the mutations located at exons 13, 20 and 29 of C4 gene, as well as the null alleles, was found to be significantly associated with SLE [20]. The same situation was also observed in Malaysian Southern Chinese by Doherty et al. [18]. However, a synergistic effect of C4 deletions and HLA-DRw15 in conferring disease susceptibility was detected.

The other complement component of particular importance in SLE is C1q. Individuals having a congenital genetic deficiency of C1q gene could develop SLE-like symptoms at more than 90% prevalence [37, 38]. Various mutations in C1q have been reported, including nonsense mutations, missense mutations and single nucleotide polymorphisms (SNP). It is conceivable that these may lead to failures in the synthesis of intact C1q molecules leading to abnormal immune responses. While C1q deficiency was reported to be associated with SLE in Turkish and Mexican subjects [39, 40], no association was observed between any of the mutations [at C1qA-Gln186 (C > T), C1qB-Gly15 (G > A), C1qB-Arg150 (C > T), C1qCGly6, (G > A), and C1qC-Arg41 (C > T)], or SNPs [at C1qAGly70 (G/A), and C1qC-Pro14 (T/C)] within C1q and SLE in the Malaysia [21].

### 2.3. Tumour Necrosis Factor 

Tumour necrosis factor (*TNF*) genes are situated at the short arm of chromosome 6. TNF proteins are a group of low-molecular-weight cytokines that mediate inflammation processes. TNF-alpha (TNF-*α*) protein, also known as cachectin, has been frequently investigated. It plays an important role in the regulation of immune cells, stimulation of apoptotic cell death, and induction of inflammation. Cytokine imbalances are believed to be drivers of certain autoimmune diseases, including SLE. The first bi-allelic *TNF-*α** gene polymorphism was reported by Wilson et al. [41], which involved a single base change from G to A at the position -308 in the promoter region of the gene. A meta-analysis study revealed that the 308-A/G functional promoter polymorphism association was inconsistent. However the risk genotype A/A and risk allele A were associated with SLE in European populations but not in Asian or African populations [42]. The other member of TNF family is TNF-beta (TNF-*β*), known as lymphotoxin. The biallelic polymorphism in intron 1 of *TNF-*β** gene is believed to influence TNF-*α* production and has been associated with SLE in both Caucasian and Asian populations [43–45].

Risk allele A of TNF-*α* − 308 was associated with SLE in Malaysian cohorts as reported by Azizah et al. [22] and Chua et al. [23], in conjunction with a significant increased frequency of A/G heterozygotes in patients. The TNF-*β* +252 polymorphism in intron 1 did not feature in SLE susceptibility [23].

### 2.4. Fc Gamma Receptors

Fc gamma receptors (Fc*γ*Rs) are present on the surface of most effector cells of the immune system and involved in mediation of phagocytosis, immune complex clearance, antibody-dependent cell-mediated cytotoxicity and stimulation of inflammatory cells [46]. 

Fc*γ*RIIa is the most widely distributed member of Fc*γ*R, and *Fc*γ*RIIA* gene may occur in two allelic forms that can cause single amino acid residue modification at position 131. Fc*γ*RIIa-R131 has a relatively lower affinity for human IgG2 that causes less ability to process and clear immune complexes effectively. Thus it was suggested as a disease susceptibility factor for SLE, as observed in a meta-analysis that involved European, African, and Asian populations [47]. However many studies actually did not show association between polymorphism of Fc*γ*RIIA and SLE susceptibility in their populations [48], including Malays and Chinese in Malaysia [24]. 

Fc*γ*RIII is encoded by two distinct but highly homologous genes: *Fc*γ*RIIIA* and *Fc*γ*RIIIB*. A SNP (T to G substitution) in *Fc*γ*RIIIA* that results in a valine (V) substitution for phenylalanine (F) at amino acid residue position 158 has been correlated with SLE in Asians [35]. As for Fc*γ*RIIIB, the polymorphism may occur as neutrophil antigen 1 (NA1) or 2 (NA2). Study by Yap and coworkers [7] showed no association between Fc*γ*RIIIB-NA polymorphism and SLE in Malay and Chinese patients in Malaysia. This was in agreement with other reports in Caucasian SLE patients. They were also able to detect a Chinese SLE patient with NA-null, which is a consequence of a Fc*γ*RIIIB gene deficiency or deletion.

### 2.5. T-Cell Receptors

CD28 and CTLA-4 are receptors on T-cell surfaces that have opposite effects on T cells. CD28 is a costimulatory molecule which is responsible for T-cell proliferation, cytokine production, and the prevention of T-cell anergy [49], whereas CTLA-4 maintains the immune response at physiological level by regulating the activity of CD28 and T-cell activation. 

Few studies have been carried out to investigate the association of *CD28* gene polymorphism with SLE susceptibility. A study performed on the Malaysian population demonstrated no association between CD28 IVS3 +17T/C SNP and SLE susceptibility, although the frequency of T allele and its corresponding homozygous was the highest among the population [25]. In contrast to CD28, there have been more reports of the association of CTLA-4 polymorphisms with SLE, both in Caucasians and Asians. CTLA-4 promoter (−1722 T/C) polymorphism and (+49 A/G) polymorphism from exon-1 were found to have their TC and GG genotypes, respectively, being significantly associated with SLE in Asian populations [35, 50]. However, polymorphisms in *CTLA-4* gene (+49A/G at exon 1, −1722T/C, −1661A/G and −318C/T at promoter sites and +6230A/G in 3′-untranslated region) were not reported to be important in Malaysian SLE patients [26].

### 2.6. Interleukins

Interleukins are a group of cytokines, the majority of which are secreted by helper T cells, monocytes, macrophages, dendritic cells, natural killer cells, and B cells. They are mainly involved in promoting the development and differentiation of T and B cells and activation of natural killer cells.

Interleukin-1 (IL-1) is a polypeptide encompassing IL-1 alpha (IL-1*α*) and IL-1 beta (IL-1*β*). *IL-1* gene is located on chromosome 2, and genes encoding IL-1*α* and IL-1*β* are in close proximity to each other. The defective production of IL-1 has been implicated in development of SLE since 1983 [51]. However, not many studies have been conducted to investigate the association between *IL-1* gene polymorphisms and susceptibility to SLE. According to Chua et al. [27], SLE patients in Malaysia are susceptible to IL-1**β** −511 C/T polymorphism, with the C allele and its corresponding homozygous exhibiting a higher risk to SLE. These findings differed from a report by Parks et al. [52] that showed T allele had more potential to confer risk of SLE in African Americans. In Taiwan, no association between IL-1**β** −511 C/T polymorphism and SLE was observed [53]. In a similar study carried out by Chua and coworkers [27], a significant correlation of another IL-1**β** polymorphism (+3954 E1/E2 in exon 5) with SLE susceptibility in Malaysian population was noted, with E1 allele rather than the E2 at higher frequency among patients. This was also the case in Columbian SLE patients but not in the Taiwanese [53, 54]. 

The secretion and activity of IL-1 are tightly counter-balanced by IL-receptor antagonist (IL-1ra), which competitively binds to the same receptor as IL-1. *IL-1RN* gene, which encodes IL-1ra, is also situated on chromosome 2. The dysregulation of IL-1 production by IL-1ra will cause abnormal inflammatory activity that leads to subsequent tissue damage, which is the characteristic pathogenesis of SLE. In as much as IL-1ra may contribute to the occurrence of SLE, many studies have been done to investigate the association of polymorphisms in *IL-1RN* gene with SLE susceptibility. Polymorphism in IL-1ra is always characterised by variable numbers of an 86-bp tandem repeat in the intron 2 that may functionally affect three potential protein binding sites: an *α*-interferon silencer A, a *β*-interferon silencer B, and an acute- phase response element [55]. The first study to correlate this polymorphism with SLE susceptibility was done on Caucasians in 1994, and carriage of IL-1RN∗2 was reported to be associated with severity rather than susceptibility to SLE [56]. In Malaysia, however, the risk allele associated with SLE susceptibility in SLE patients was IL-1RN∗1 instead. The IL-1RN∗2 allele displayed an inverse association [28]. 

Interleukin-4 (IL-4) is secreted by T-helper type-2 cells and responsible for proliferation and differentiation of B and T cells, as well as production of antibodies. *IL-4* gene is located on human chromosome 5, and the study of the impact of its polymorphisms on SLE susceptibility is not as popular as other candidate genes. An IL4 haplotype −590C/−33C/9241G/14965C was significantly associated with SLE in Taiwan Chinese population [57]. Another study in Taiwan revealed the association of IL-4 −590T/C and intron 3 VNTR (variable number of tandem repeats) polymorphisms with the presence of certain clinical manifestations in SLE patients [58]. In the Malaysian cohort that was studied, the VNTR variants within intron 3 of *IL-4* gene were not associated with SLE susceptibility [8].

Interleukin-6 (*IL-6*) gene, located on chromosome 7, is another interleukin gene of interest that has been studied and associated with the susceptibility of SLE. IL-6 promoter polymorphism (−174 G/C) is commonly investigated as a risk factor in SLE. While a study on Malaysian population found a significant correlation between homozygous G genotype and SLE susceptibility, none of the studies in Taiwan, Iran, and Portugal reported the association of this polymorphism with SLE in their populations [29, 59–61]. 

Interleukin-10 is also believed to play an important role in the pathogenesis of SLE. Various polymorphisms in IL-10 promoter region have been reported to display significant association with SLE susceptibility [62–64]. An earlier study reported that the IL10.G microsatellite alleles in IL-10 promoter region had significantly higher frequency in Caucasian SLE patients [64]. A study investigating the relationship of three SNPs in *IL-10* gene promoter (−1082G > A, −824C > T, and −597C > A) with SLE susceptibility in Malaysian population revealed that haplotype frequencies rather than genotypes or alleles were more important [30].

### 2.7. Other Genes

The role of angiogenic-converting enzyme (*ACE*) gene I/D dimorphism in susceptibility to SLE in the Malaysian population was illustrated by Lian and coworkers [31]. *ACE* gene, located on the q arm of chromosome 17, produces protein which is an important player in the renin-angiotensin system and kallikrein-kininogen system [65]. Dysregulation of ACE could lead to vascular damage, particularly in kidneys of SLE patients. In that study, no significant difference was observed in the distribution of I and D alleles between cases and healthy controls although ID heterozygote did show significant association with SLE [31]. This finding was in accordance with what was reported in African-American and European-American populations [66] but contradicted those in Japanese and Slovakian populations, whereby I and D alleles were found, to be significantly associated with SLE, respectively [66, 67].

Polymorphisms at position 28 of the regulated on activation, normal T cell expressed and secreted (*RANTES*) gene promoter region and position 801 in 3′ UTR of stromal cell-derived factor 1 (*SDF-1*) gene were also analysed by Lian et al. [32]. Again, both polymorphisms did not show significant association with SLE in Malaysia and similar observations were also reported in Mexican and Han Chinese populations [68–70].

## 3. Conclusion

Most studies conducted in Malaysian SLE patients did not exhibit significant association of the candidate genes with susceptibility, safe for a few which are within the human MHC. There are several pertinent reasons for these findings. Firstly, this could be due to smaller sample sizes as it is often difficult to obtain large numbers of SLE patients within a medical centre or hospital within a relatively short period of time when such studies are undertaken. So far, there has yet to be a long term or longitudinal national study on this enigmatic disease. The second reason may be that given the complexity of SLE and the dynamic nature of the disease, there may well be different sets of genes and biological players that assume various roles during the precipitation and pathogenesis of SLE from predisposition to actual onset and resultant progression. The genetic heterogeneity evident in different SLE patients of various ethnicities could also be attributed to the inheritance of different ancestral genotypes that impact upon the development and/or progression of this disease [13]. Gene-gene and gene-environment interactions could also confer differences in susceptibility to or be protective against a particular disease in different populations or ethnic groups. It is hoped that with larger and better defined patient sets and appropriate controls, more comprehensive genetics and systems biology approaches, and better technologies, we will be able to gain a better understanding of SLE and insight into ways of managing this most enigmatic and challenging of autoimmune diseases. 

## Figures and Tables

**Table 1:** Summary of associations between candidate genes and SLE susceptibility in the Malaysian population.

Gene	Ethnicity	Cases	Controls	Allotype/minor allele frequency		*P *value	RR/OR	Reference
				Cases	Controls			
HLA								
DR2	Malays	56	59	48 (85.7%)	36 (61%)	0.03^#^	3.83	[16]
DQB1∗0501	Malays	56	59	27 (48.2%)	10 (16.9%)	0.0036^#^	4.56	[16]
	Chinese	70	66	19 (27.1%)	5 (7.6%)	0.003^#^	4.55	[17]
DQB1∗0601	Malays	56	59	20 (35.7%)	5 (8.5%)	0.0048^#^	6.00	[16]
	Chinese	70	66	28 (40%)	9 (13.6%)	0.006^#^	4.22	[17]
DQA1∗0102	Chinese	70	66	61 (43.6%)	44 (33.3%)	0.032^#^	3.39	[17]
DPB1∗0901	Chinese	70	66	22 (31.4%)	6 (9.1%)	0.02^#^	4.58	[17]
DRw15	Chinese	87	66	37.9%	10.6%	<0.006^#^	5.2	[18]
DQw1	Chinese	88	63	75.0%	57.1%	<0.006^#^	5.2	[18]
A∗1101, 1102	Malaysian	160	107	33.05%	18.69%	0.0002^#^	2.147	[19]
DRB5∗0101, 0102, 0201, 0202, 0203	Malaysian	160	107	56.52%	41.90%	0.0014^#^	1.802	[19]
DRB3∗0101, 0201, 0202, 0203, 0301	Malaysian	160	107	42.03%	71.43%	0.000^#^	0.290	[19]
DQB1∗05	Malaysian	160	107	37.80%	20.61%	0.0000^#^	2.341	[19]
DQB1∗0301, 0304	Malaysian	160	107	12.80%	32.89%	0.0000^#^	0.300	[19]
DRB1∗0701	Malays	61	49	14.13%	4.76%	0.0356^#^	3.291	[19]
DRB4∗0101101, 0102, 0103 (not DR53N)	Malays	61	49	47.83%	19.05%	0.0001^#^	3.896	[19]
DRB1∗1601-1606	Chinese	99	58	14.46%	3.79%	0.0030^#^	4.090	[19]
DRB5∗0101, 0102, 0201, 0202, 0203	Chinese	99	58	51.81%	34.92%	0.0040^#^	2.003	[19]

C4								
2bp insertions (+TC) at codon 1213 in exon 29	Malaysian	130	130	0	0	—	—	[20]
1bp deletions (−C) at codon 811 in exon 20	Malaysian	130	130	0	0	—	—	[20]
1bp deletion (−C) at codon 522 in exon 13	Malaysian	130	130	0	0	—	—	[20]
2bp deletions (−GT) at codon 497 in exon 13	Malaysian	130	130	0	0	—	—	[20]
Null alleles	Malaysian	130	130					[20]
C4A∗Q0				0	2	—	—	
C4B∗Q0				2	0	—	—	
C4A gene with long C4B gene	Chinese	85	63	57.6%	68.3%	—	—	[18]
C4A gene with short C4B gene	Chinese	85	63	67.1%	76.2%	—	—	[18]
C4A or C4B gene deletion	Chinese	85	63	34.1%	27.0%	—	—	[18]
C4X	Chinese	85	63	41.2%	28.6%	—	—	[18]

C1q								
C1qA-Gln186 (C > *T*)	Malaysian	130	130	0	0	—	—	[21]
C1qB-Gly15 (G > *A*)	Malaysian	130	130	0	0	—	—	[21]
C1qB-Arg150 (C > *T*)	Malaysian	130	130	0	0	—	—	[21]
C1qC-Gly6 (G > *A*)	Malaysian	130	130	0	0	—	—	[21]
C1qC-Arg41 (C > *T*)	Malaysian	130	130	0	0	—	—	[21]
C1qA-Gly70 (G/A)	Malaysian	130	130	47	45	0.660	1.081	[21]
C1qC-Pro14 (T/C)	Malaysian	130	130	75	79	0.254	0.789	[21]

TNF								
TNF-*α* −308 G/A	Chinese	70	59	37	20	0.003^#^	1.42	[22]
	Malaysian	100	100	42	22	0.0064^#^	2.1507	[23]
TNF-*β* +252 A/G	Malaysian	100	100	117	111	0.5446	1.1303	[23]

Fc*γ*R								
Fc*γ*RIIA (H131R)	Chinese	175	108	0.40	0.45	0.3200	0.83	[24]
	Malays	50	50	0.34	0.37	0.7676	0.88	[24]
Fc*γ*RIIIB (NA1 or NA2)	Chinese	183	100	0.347	0.32	—	—	[7]
	Malays	55	50	0.38	0.38	—	—	[7]

CD28								
IVS3 +17 T/C	Malaysian	100	100	46 (23)	41(20.5)	0.5446	1.1584	[25]

CTLA-4								
Exon 1 (+49 A/G)	Malaysian	130	130	155 (0.60)	151 (0.58)	0.722	0.94	[26]
Promoter site (−1722 T/C)	Malaysian	130	130	90 (0.35)	103 (0.40)	0.238	0.81	[26]
Promoter site (−1661 A/G)	Malaysian	130	130	27 (0.10)	37 (0.14)	0.182	1.43	[26]
Promoter site (−318 C/T)	Malaysian	130	130	19 (0.07)	18 (0.07)	0.865	1.06	[26]
3′-UTR (+6230 A/G)	Malaysian	130	130	34 (0.13)	22 (0.09)	0.117	1.63	[26]

IL								
IL-1*β* −511 C/T	Malaysian	100	100	96	139	<0.05^#^	0.4051	[27]
IL-1*β* +3954 E1/E2	Malaysian	100	100	49	77	<0.05^#^	0.5184	[27]
IL-1RN	Malaysian	100	100					[28]
IL-1RN∗1				196 (96%)	180 (90%)	0.019^#^	2.667	
IL-1RN∗2				6(3%)	18 (9%)	0.012^#^	0.313	
IL-4 third intron RPI/RPII	Malaysian	100	100	54 (27%)	55 (27%)	0.9106	0.9751	[8]
IL-6 −174 G/C	Malaysian	100	100	53 (26.5%)	95 (47.5%)	0.0000136^#^	0.3985	[29]
IL-10 −1082 G/A	Malaysian	44	44	8 (9%)	12 (13%)	0.342	—	[30]
IL-10 −824 C/T	Malaysian	44	44	55 (62.5%)	53 (60%)	0.757	—	[30]
IL-10 −597C/A	Malaysian	44	44	55 (62.5%)	53 (60%)	0.757	—	[30]

ACE I/D dimorphism	Malaysian	170	190	117 (34.4%)	138 (36.8%)	0.5938	0.9201	[31]
RANTES-28 C/G	Malaysian	130	130	14	19	0.3684	0.7219	[32]
SDF-1 3′ UTR G801A	Malaysian	130	130	116	132	0.1601	0.7811	[32]

^#^-significant association, RR: relative risk, OR: odds ratio, ^—^not studied.
